# Sulfatide Inhibits HMGB1 Secretion by Hindering Toll-Like Receptor 4 Localization Within Lipid Rafts

**DOI:** 10.3389/fimmu.2020.01305

**Published:** 2020-06-23

**Authors:** Hee Sue Kim, Myeonggil Han, In Ho Park, Cheol Ho Park, Man Sup Kwak, Jeon-Soo Shin

**Affiliations:** ^1^Department of Microbiology, Yonsei University College of Medicine, Seoul, South Korea; ^2^Brain Korea 21 PLUS Project for Medical Science, Yonsei University College of Medicine, Seoul, South Korea; ^3^Severance Biomedical Science Institute, Yonsei University College of Medicine, Seoul, South Korea; ^4^Institute for Immunology and Immunological Diseases, Yonsei University College of Medicine, Seoul, South Korea

**Keywords:** sulfatide, HMGB1, TLR4, lipid raft, sepsis, NF-κB, ROS

## Abstract

The high mobility group box 1 (HMGB1) is a well-known late mediator of sepsis, secreted by multiple stimuli, involving pathways, such as the mitogen-activated protein kinase (MAPK) and nuclear factor kappa B (NF-κB) pathways, and reactive oxygen species (ROS) under inflammation. Sulfatide, in contrast, is a sphingolipid commonly found in myelin sheets with a disputed immunological role. We sought to determine the immunological characteristics of sulfatide in the periphery by analyzing the secretion of HMGB1 triggered by lipopolysaccharide (LPS) stimulation in Raw 264.7 cells. Suppression of HMGB1 secretion by inhibiting its cytosolic translocation was observed after pre-treatment with sulfatide before LPS stimulation. Further analysis of the downstream molecules of toll-like receptor (TLR) signaling revealed suppression of c-Jun N-terminal kinase (JNK) phosphorylation and p65 translocation. LPS-mediated ROS production was also decreased when sulfatide pre-treatment was provided, caused by the down-regulation of the phosphorylation of activators, such as IRAK4 and TBK1. Investigation of the upstream mechanism that encompasses all the aforementioned inhibitory characteristics unveiled the involvement of lipid rafts. In addition to the co-localization of biotinylated sulfatide and monosialotetrahexosylganglioside, a decrease in LPS-induced co-localization of TLR4 and lipid raft markers was observed when sulfatide treatment was given before LPS stimulation. Overall, sulfatide was found to exert its anti-inflammatory properties by hindering the co-localization of TLR4 and lipid rafts, nullifying the effect of LPS on TLR4 signaling. Similar effects of sulfatide were also confirmed in the LPS-mediated murine experimental sepsis model, showing decreased levels of serum HMGB1, increased survivability, and reduced pathological severity.

## Introduction

Approximately 45 years have passed since the HMGB1 protein, an abundant nuclear protein and a well-defined danger-associated molecular pattern (DAMP) molecule, was first purified (1). Since its discovery, HMGB1 has been discussed in various contexts. Nuclear HMGB1 is well-known for its chaperone-like functions, playing a role in deoxyribonucleic acid (DNA) unwinding (2) and DNA synthesis (3) by binding to DNA in a sequence-independent manner (4) and in the structuring of chromatin (5). In contrast, research regarding cytosolic HMGB1 is still in its relatively early stages, revealing its role in autophagy regulation (6) and unconventional protein secretion (7).

HMGB1 can be either passively released through non-apoptotic cell death, such as in necrotic cells (8), or actively secreted through multiple pathways, such as in inflammasome-mediated release (9). In this paper, we intend to limit the scope to active secretion of HMGB1, triggered by inflammatory signals transduced by toll-like receptor (TLR)-related signaling. When TLRs are stimulated by their ligands, NF-κB (10) and MAPK (10–12) are responsible for the translocation and secretion of these receptors to the extracellular space. Our research concentrates on TLR4, a member of the TLR family, which recognizes lipopolysaccharides (LPS), and its mechanism of action regarding the active secretion of HMGB1. Under physiological conditions, bacterial LPS, which normally forms a micelle, is recognized by the LPS-binding protein (LBP), which facilitates its monomerization by CD14 (13). The LPS–LBP complex, now bound to CD14, is then transferred to the myeloid differentiation protein-2 (MD-2)–TLR4 complex (14). This complex then forms a dimer, completing its activation process. TLR4 dimers, however, require the formation of a lipid raft, a special nano-scale membrane structure consisting of various lipids (15). TLR4, which contains lipid-binding motifs, is attracted and can readily form a dimer within the lipid rafts, providing a platform on which the TLR4s can be within closer proximity (16).

Most studies addressing the immunological role of HMGB1 have focused on the role of extracellular HMGB1 as a DAMP molecule and its chemokine-like behavior. Depending on its redox status, HMGB1 exerts different characteristics: 1) as a thiol isoform, in which all of the three active cysteine residues (Cys23, 45, and 106) are in free-thiol(-SH) form, HMGB1 binds to C-X-C motif ligand 12 (CXCL12) and shows chemokine-like activity, recruiting immune cells to the site of inflammation (17); 2) the disulfide isoform of HMGB1, which possesses one intra-molecular disulfide bond between the two cysteine molecules Cys23 and Cys45, exerts cytokine-like activity, activating macrophages and lymphocytes (18–20); 3) the oxidized isoform of HMGB1, containing fully oxidized cysteine residues (-SOOOH) is considered immunologically inert (21, 22). In sepsis, extracellular HMGB1 is known to be released in its reduced form (23); it is considered a potent pro-inflammatory cytokine (24) and a promising therapeutic target in clinical studies (25, 26).

Sulfatide, also known as 3-O-sulfogalactosylceramide, is a lipid commonly found in the myelin sheath in both the central and peripheral nervous system (27). First isolated and partially characterized over 40 years ago (28), sulfatide was suggested to play a varying role in physiological functions, ranging from myelination of nerves (29, 30) to insulin secretion (31–34). Similar to HMGB1, intracellular (or membrane-bound) sulfatide and extracellular sulfatide play different roles. While the intracellular (or membrane-bound) form performs the abovementioned functions, extracellular sulfatide can bind to selectins to cause hemostasis (35) or metastasis of tumors (36) or bind to CD1d activating natural killer T (NKT) cells with various anti-inflammatory abilities (37–41). Although most papers discussing the anti-inflammatory functions of sulfatide emphasize on NKT cells, a report suggested that sulfatide may have a direct effect on brain-resident immune cells, causing inflammation (42). This discrepancy between immune cells residing in the central or peripheral nervous system led us to investigate the direct effect of sulfatide in peripheral immune cells, namely the macrophages.

In this study, we aimed to elucidate the effect of sulfatide in the context of innate immunity by investigating its effect on HMGB1 secretion under LPS stimulus and discuss the specific molecules involved in the process.

## Materials and Methods

### Cell Culture and Treatment Reagents

Raw 264.7 cells were cultured in Dulbecco's Modified Eagle's Medium supplemented with 10% heat-inactivated fetal bovine serum (Gibco, Waltham, MA, USA), 100 μg/mL of penicillin, and 100 μg/mL of streptomycin (Sigma, Saint Louis, MO, USA). Treatment was performed after allowing the cells to adapt to Opti-MEM (Gibco) for 2 h, after which the media was replaced.

LPS (*Escherichia coli* O111:B4; > 3 x 10^6^ EU/mL; Sigma), sulfatide (Bovine; brain; Matreya, State College, PA, USA), 18:0(2R-OH) sulfogalactosylceramide (synthetic; Avanti, Alabaster, AL, USA), C24:0 mono-sulfogalactosylceramide (synthetic; Avanti), C24:0 mono-sulfogalactosylceramide (synthetic; Avanti), galactosylceramide (Bovine; Matreya), and ceramide (Bovine; Matreya) were used as indicated in the figures. All experiments were performed using vehicle as a negative control.

### Bone Marrow-Derived Macrophage (BMDM) Preparation

Wild-type C57BL/6 mice obtained from Orient Bio (Seongnam, Gyeonggi-do, South Korea) were housed in a SPF-grade facility with controlled temperature, humidity, and light. For all experiments, 8-week old female mice with approximate body weight of 20 g were used. The animals were ethically sacrificed, and the femur and tibia were extracted. Bone marrow was collected via warm, serum-free DMEM lavage until no remaining bone marrow was visible. Bone marrow was collected and filtered through cell strainer with 40 μm pore (SPL, Pocheon-si, Gyeonggi-do, South Korea) to remove any undesirable debris and washed with excessive media to further remove unfiltered debris. The resulting cells were plated to 100 mm cell culture-treated dish (Corning, Oneonta, NY, USA), and then differentiated using 20 ng/mL GM-CSF in complete medium for 7 days to yield BMDMs.

### Sample Preparation (Culture Media)

Culture media after treatment were collected after 24 h to compare HMGB1 secretion between groups. Culture media were then centrifuged at 3500 × g for 5 min to remove any cell debris. The supernatant was collected for trichloroacetic acid (TCA)/acetone precipitation. Then, 10% by volume of ice-cold TCA was added to the samples and mixed by inverting. After incubating overnight at−20°C, the samples were thawed and centrifuged at 20000 × g for 90 min. Supernatants were then discarded. The remaining pellets were washed with−20°C acetone by vortexing vigorously and left overnight at−20°C. Samples were centrifuged at 20000 × g for 90 min, and the resulting supernatants were removed. The remaining pellets were then dried and boiled with 2X sample buffer.

### Sample Preparation (Whole Cell Lysate)

Cells were harvested by scraping using cold Dulbecco's phosphate buffered saline (PBS) after the indicated time periods; they were then collected by centrifuging at 3000 × g for 5 min. Supernatants were discarded, and radioimmunoprecipitation (RIPA) buffer was added before sonication. Lysed cells were centrifuged at 20000 × g for 10 min to remove any debris. The resulting whole cell lysates were collected, and protein concentration was quantified using the bicinchoninic acid (BCA) assay. The cell lysates were then prepared by heating to 65°C for 10 min after adding sample buffer to minimize the loss of phosphorylated protein to beta-elimination.

### Western Blot

SDS-PAGE was performed on samples prepared via the abovementioned methods, and proteins were transferred to a polyvinylidene difluoride (PVDF) membrane for western blotting. Transferred membranes were blocked using 5% skimmed milk. Primary antibodies for HMGB1 (Abcam; Cambridge, UK), JNK (phospho- and whole; Cell Signaling Technology; Danvers, MA, USA), ERK1/2 (phospho- and whole; Cell Signaling Technology), p38 (phospho- and whole; Cell Signaling Technology), phospho-IκBα (Cell Signaling Technology), phospho-IRAK4, phospho-TBK1 (Cell Signaling Technology), caveolin 1 (Merck; Darmstadt, Germany), TLR4 (Santa Cruz; Dallas, TX, USA), and β-actin (Santa Cruz) were diluted in 5% skimmed milk solution and incubated overnight at 4°C. After extensive washing, the corresponding secondary antibody solutions were incubated for 1 h at room temperature (20~25°C). The membranes were then washed, and signals were detected using enhanced chemiluminescence substrate solution (Gendepot; Katy, TX, USA) and X-ray film (AGFA; Mortsel, Belgium). Membranes were stripped using stripping solution (BioMax, Seoul, South Korea) for re-blotting, as necessary. Densitometry analysis was performed using Image J.

### Immunofluorescence

Raw 264.7 cells were seeded in 4-well chambered glass slides coated with poly-L-lysine (Sigma). Treatment dosage for LPS was increased to 200 ng/mL to facilitate visualization via immunofluorescence, and sulfatide dosage was adjusted accordingly to maintain molar ratio. Treatment was performed for the duration indicated in Figure Legends. After treatment, cells were then fixed with 4% paraformaldehyde overnight in 4°C. On the subsequent day, the cells were washed with PBS and permeabilized with 1% Triton X-100 and blocked with bovine serum albumin (BSA). Primary antibodies anti-p65 (Santa Cruz) or anti-HMGB1 (Abcam) were diluted in BSA solution and left to incubate overnight at 4°C. After thorough washing, the respective secondary antibodies conjugated with Alexa Fluor 488 (Invitrogen; Waltham, MA, USA) were diluted in BSA solution and incubated at 37°C for 45 min. Slides were then washed, dried, and mounted using mounting medium containing 4′,6-diamidino-2-phenylindole (DAPI; Vector). Sealed slides were observed via FV1000 confocal microscopy (Olympus). Localization of sulfatide was determined by treating Raw 264.7 cells with biotin-sulfatide and staining them with streptavidin-Alexa Fluor 488 (Invitrogen). Localization of TLR4 was detected using mouse anti-TLR4 antibodies (Invitrogen).

### ROS Detection

Raw 264.7 cells were pre-treated with either vehicle control or 20 μM of sulfatide and with vehicle control or 100 ng/mL of LPS for 1 h. The treatment medium was removed, and culture dishes were washed twice with warm culture medium. H2-DCFDA (Thermo Fisher; Waltham, MA, USA) was treated as per the manufacturer's instructions. Cells were viewed under a fluorescence microscope. For flow cytometric analysis of ROS levels, the cells were detached before H2-DCFDA treatment.

### Lipid Raft Staining

Raw 264.7 cells were seeded in 4-well chambered glass slides coated with poly-L-lysine (Sigma). Treatment dosage for LPS was increased to 1 μg/mL to maximize lipid raft formation and facilitate visualization via immunofluorescence, and sulfatide dosage was adjusted accordingly to maintain molar ratio. Treatment was performed for the duration indicated in Figure Legends. After treatment, the cells were then washed once with 4°C complete growth medium. Washed cells were incubated in cholera toxin B-Alexa Fluor 549 (Invitrogen) staining solution, prepared in 4°C complete growth medium. Cells were washed with ice-cold PBS three times and fixed with ice-cold 4% paraformaldehyde for 15 min.

### Lipid Raft Isolation

Raw 264.7 cells were treated with reagents for 8 min as indicated in the legends, and cells were briefly washed three times with ice-cold PBS to halt the internalization of lipid rafts. Cells were then lysed using the ice-cold buffer provided by Caveolea/Rafts Isolation Kit (Merck) supplemented with Triton X-100. Lysates then underwent ultracentrifugation with OptiPrep™ density gradient, provided by the aforementioned kit, and nine fractions were collected. Collected fractions were then supplemented with 1% SDS to assist complete dissociation of the protein from the lipids. Treated samples were concentrated using TCA/Acetone and analyzed by immunoblotting.

### Animal Experiments

Wild-type C57BL/6 mice obtained from Orient Bio (Seongnam, South Korea) were housed in a SPF-grade facility with controlled temperature, humidity, and light. For all experiments, 8-week old female mice were used. For serum collection, mice were anesthetized using an isoflurane–oxygen mixture, and combinations of PBS, LPS (3 mg/kg), or sulfatide (25 nmol) were injected with a total of 100 μL injection volume, intraperitoneally. The animals were allowed 60 min between injections to fully recover from the effects of anesthesia. Serum samples were collected after 18 h. Survival rate was measured by following the same procedure as mentioned above, with increased doses of LPS and sulfatide injection (to 20 mg/kg and 175 nmol, respectively). Mice were checked twice every day and observed until completion. Survival data were then analyzed through Kaplan-Meier survival analysis. Pathological scores were obtained using the scoring regimen described by Shrum et al. (43), and the obtained scores were then analyzed through ANOVA and Dunnett's multiple comparison test. All experiments were conducted according to procedures approved by the Institutional Animal Care and Use Committee of the Yonsei Laboratory Animal Research Center (YLARC, 2015-0275).

### Enzyme-Linked Immunosorbent Assay (ELISA)

Tumor necrosis factor α (TNF-α) and interleukin-6 (IL-6) ELISA were performed using Raw 264.7 cell culture medium. Cells were treated with vehicle control (negative control), 100 ng/mL LPS, and sulfatide 20 μM, followed by LPS 100 ng/mL. Culture media were collected after 12 h of treatment and centrifuged to remove any cell debris. ELISA was performed with the resulting supernatant following the manufacturer's instructions (Invitrogen). Serum obtained from murine experimental sepsis models was analyzed for HMGB1 levels with a HMGB1 ELISA kit (IBL International), following the manufacturer's instructions.

### Statistical Analysis

Unless specified otherwise, statistical analysis of experimental data present in this paper were performed with Student's t test and ANOVA, with Tukey's multiple comparison test as *post-hoc* test, using GraphPad Prism 5. The data represent the mean value and SD. The difference was considered statistically significant at *p* < 0.05.

## Results

### Sulfatide Inhibits HMGB1 and Pro-inflammatory Cytokines Release

**Figure 1:**
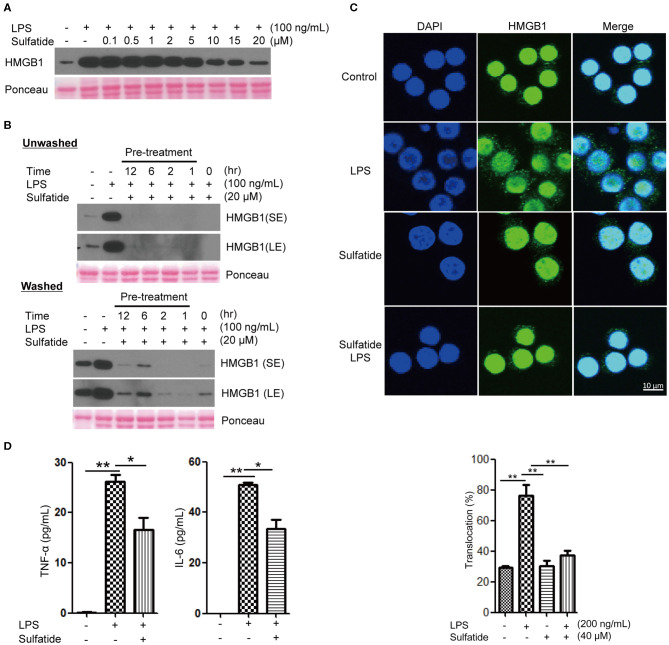
Sulfatide inhibits HMGB1 translocation and release in Raw 264.7 cells. **(A)** Dose-dependency of sulfatide regarding HMGB1 secretion was accessed 24 h after LPS treatment. Varying dosage of sulfatide was treated 10 min prior to LPS treatment. **(B)** Efficacy of sulfatide pre-treatment for indicated time on HMGB1 secretion and its effect after removal of sulfatide was observed. Washed cells received two 36°C PBS wash to remove the residual sulfatide prior to LPS treatment, whereas unwashed cells were left unperturbed. (SE, Short Exposure; LE, Long Exposure) **(C)** Raw 264.7 cells received vehicle control, LPS 200 ng/mL, sulfatide 40 μM only, or 10 min of sulfatide 40 μM pre-treatment, followed by LPS 200 ng/mL for 6 h. Cells were fixed for analysis by immunofluorescence, as described in the Methods section. In total, 100 cells were counted, and those containing HMGB1 signals in the cytoplasm were counted as positive. **(D)** Culture media were analyzed by ELISA for TNF-α and IL-6 titer. Cells were treated with vehicle control (PBS with DMSO), LPS 100 ng/mL, or 10 min of sulfatide 20 μM pre-treatment, followed by LPS 100 ng/mL. Graphs show the mean value and error bars of three independent experiments performed. *p < 0.01, **p < 0.001.

To study whether sulfatide treatment shows pro-inflammatory or anti-inflammatory characteristics, we analyzed the secretion level of a well-known DAMP molecule, HMGB1. When treated simultaneously, sulfatide exhibited an inhibitory effect in HMGB1 secretion without toxicity in a dose-dependent manner, as shown (Figure 1A, Supplementary Figure 1A). This phenotype was unique to sulfatide, and was not seen in its precursors, galactosylceramide, and ceramide (Supplementary Figure 1B). Further analysis using ligands of other extracellular TLRs shows complete inhibition of HMGB1 secretion (Supplementary Figure 1C). This indirectly suggests that the anti-inflammatory effect does not come from inhibiting the ligand-receptor interaction by acting as a competitive inhibitor or aggregating reagent against TLR ligands, since it is unlikely that a molecule can act as broad-range inhibitor or aggregating reagent against multiple TLR ligands with different characteristics. Next, the time point-dependent effect of sulfatide was studied to further investigate the mechanism of action (Figure 1B). Interestingly, sulfatide not only exhibited dose- and time-dependent manner in HMGB1 release suppression, but also removal of sulfatide only induced a slight increase—lower than the secretion level of negative control, nevertheless—in HMGB1 secretion in 6 and 12 h-pretreatment samples. These results, combined with the results collected above, suggest that sulfatide is neither an aggregating reagent nor competitive inhibitor, nor a reversible non-competitive inhibitor of TLR ligands.

Although multiple points of inhibition are potentially available throughout the HMGB1 secretion pathway, they can be categorized into two large categories: initial signal transduction, and the release step. In order to clarify whether sulfatide affects the former or the latter, we treated Raw 264.7 cells with LPS or sulfatide and investigated HMGB1 localization via immunofluorescence microscopy (Figure 1C). Confocal microscopy images show sulfatide inhibits nuclear HMGB1 translocation to the cytoplasm caused by LPS stimulation. This indicates that the inhibition mechanism of sulfatide does not target the release of HMGB1 to the extracellular space itself, but the pathway that precedes HMGB1 translocation.

Previous reports state sulfatide to play a pro-inflammatory role in brain-resident immune cells (42). In order to confirm its anti-inflammatory characteristics shown within our experimental setup, we treated Raw 264.7 cells with vehicle control, LPS alone, or LPS stimuli after sulfatide pre-treatment (Figure 1D). Contrary to previous reports made with brain-resident immune cells, sulfatide did not induce any significant secretion of pro-inflammatory cytokines, namely TNF-α and IL-6. Interestingly, a combination of LPS and sulfatide, however, did result in a significant decrease in the secretion levels of both TNF-α and IL-6, indicating that sulfatide indeed has an anti-inflammatory role in the peripheral immune system.

### Sulfatide Down-Regulates NF-κB Signaling Pathway and JNK Phosphorylation

**Figure 2:**
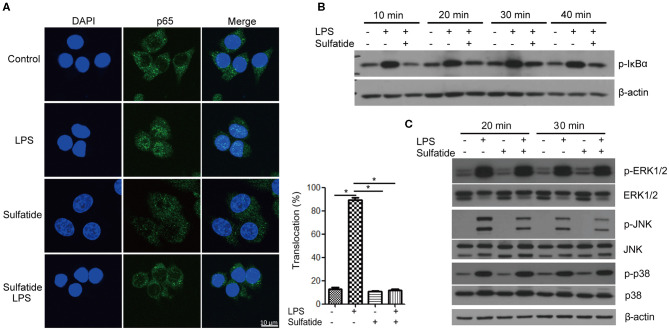
Sulfatide suppresses NF-κB activation and JNK phosphorylation. **(A)** Raw 264.7 cells received vehicle control, LPS 200 ng/mL, sulfatide 40 μM, or 10 min of 40 μM sulfatide pre-treatment, followed by LPS 200 ng/mL for 40 min. Cells were then fixed for analysis by immunofluorescence as described in the Methods section. In total, 100 cells were counted, and those with p65 signals co-localizing with DAPI were counted as positive. *p < 0.001. **(B,C)** Raw 264.7 cells received vehicle control, LPS 100 ng/mL, sulfatide 20 μM, or 10 min of 20 μM sulfatide pre-treatment followed by LPS stimuli, as shown in the figure. Cells were harvested after the indicated times and analyzed for the phosphorylation level of IκBα **(B)**, p-ERK, p-JNK, and p-p38 **(C)** by immunoblotting.

The pathway most frequently associated with TLR signaling, NF-κB signaling pathway, is a cascade of signaling molecules that results in the degradation of NF-κB inhibitory molecules and the translocation of NF-κB to the nucleus, acting as a transcription factor. Concerning this pathway, we performed immunofluorescence microscopy, tracking the location of the p65 molecule, and immunoblotting of the IκBα molecule (Figures 2A,B). Our immunofluorescence data shows that NF-κB activation, signified by the translocation of p65, decreased when cells were pre-treated with sulfatide. Immunoblotting also indicated that phosphorylation of IκBα, a crucial step that precedes its ubiquitination and degradation, significantly decreases when pre-treated with sulfatide.

Further analysis of the MAPKs within the TLR signaling pathway revealed specific kinases affected by sulfatide treatment. The phosphorylation levels of ERK, JNK, and p38 MAPK were analyzed via immunoblotting (Figure 2C). Immunoblots revealed that only the phosphorylation level of JNK, but not of ERK or p38, was decreased by pre-treatment with sulfatide. Overall, sulfatide blocks the NF-κB signaling pathway and JNK-mediated HMGB1 translocation.

### LPS-Mediated ROS Production Is Decreased by Sulfatide

**Figure 3:**
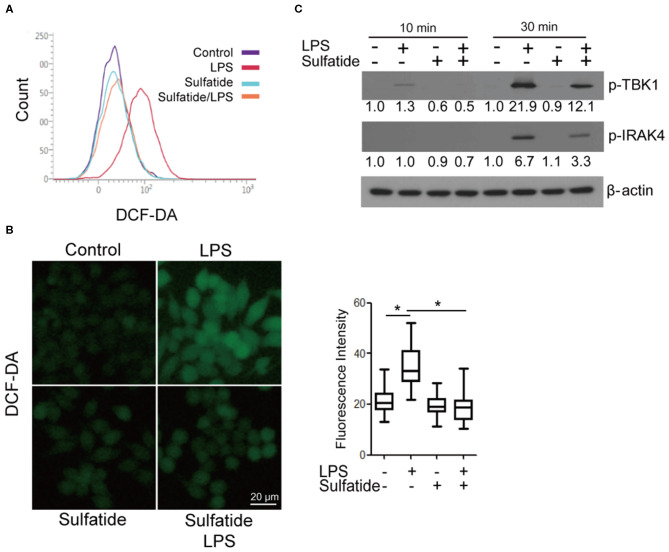
Sulfatide down-regulates LPS-induced ROS production. Raw 264.7 cells were treated with 20 μM sulfatide or vehicle control for 10 min prior to receiving LPS 100 ng/mL, and were analyzed through **(A)** flow cytometry and **(B)** fluorescence microscopy. More than 150 cells were counted. *p < 0.001. **(C)** Cells were pre-treated with vehicle control or 20 μM sulfatide before treatment with 100 ng/mL LPS. Cells were lysed, and the samples were immunoblotted for p-TBK1 and p-IRAK4. Numbers below the immunoblots represent the relative band intensity, obtained by densitometry analysis. Vehicle controls of each groups were considered as standards.

Since an alternate mechanism exists, where HMGB1 release can be triggered via LPS-TLR4 signaling through ROS production, we sought to measure the changes in the level of intracellular ROS in the presence/absence of sulfatide pre-treatment (Figures 3A,B). Flow cytometric analysis and measurement of relative fluorescence intensity both show a significant decrease in intracellular ROS levels in sulfatide pre-treated groups. Such a decrease in ROS levels can be accredited to the decreased phosphorylation of both TBK1 and IRAK4, molecules that play crucial roles in the regulation of NOX activity (Figure 3C). These results, paired with those presented in earlier experiments, propose that the point of inhibition, which sulfatide utilizes to suppress HMGB1 release is positioned higher in the signaling hierarchy.

### Sulfatide Hinders the Translocation of TLR4 Into Lipid Rafts

**Figure 4:**
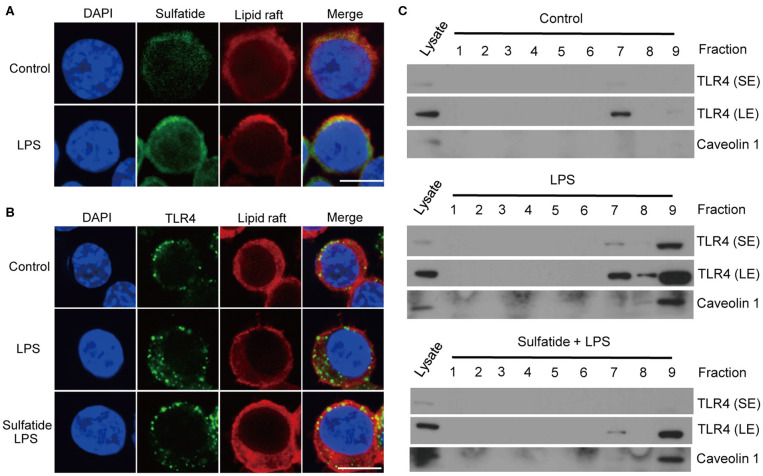
TLR4–lipid raft complex formation is reduced by sulfatide. **(A)** Raw264.7 cells were treated with 200 μM of biotinylated sulfatide, with or without 1 μg/mL of LPS for 8 min. Biotinylated sulfatide was stained with streptavidin-Alexa Fluor 488 and TLR4 with anti-rabbit-Alexa Fluor 549, and the cells were prepared for confocal microscopy as described in the Methods section. (Scale bar : 10 μm) **(B)** Raw 264.7 cells were treated with vehicle control or 40 μM of sulfatide for 10 min, before 8 min of vehicle control or 200 ng/mL of LPS treatment. **(C)** Raw 264.7 cells were identically treated as those in **(B)**, and obtained samples were immunoblotted for TLR4 and caveolin 1. All membranes were immunoblotted under identical medical X-ray film for accurate comparison. (SE, Short Exposure; LE, Long Exposure).

We hypothesized that sulfatide, a well-known component of the cell membrane, may interfere with the lipid composition of the cell membrane, inhibiting its signaling pathways. Since TLR4 requires its monomers to be localized within the lipid raft microdomains to form dimers, we sought to assess (1) whether sulfatide localizes to the lipid raft microdomains, and (2) whether sulfatide treatment curbs the localization of TLR4 to lipid rafts. Utilization of biotinylated sulfatide revealed the co-localization of sulfatide and lipid rafts (Figure 4A), indicating the possibility of direct involvement of sulfatide in the lipid raft machinery. Next, to observe the co-localization of TLR4 and lipid rafts, we treated cells with appropriate stimuli and were prepared for immunofluorescence. Results insinuated that sulfatide plays a role in significantly decreasing the localization of TLR4 into the lipid microdomains. Such findings were reinforced by subjecting the cells to identical conditions and fractionating the cell lysate for lipid rafts. Results showed significantly decreased co-localization of TLR4 within the lipid raft fractions, signified by caveolin-1, in sulfatide-treated groups. In summary, sulfatide was found to interfere with the localization of TLR4 within lipid rafts, decreasing the efficacy of TLR4 signaling (Figures 4B,C).

### Relase of HMGB1 Is Suppressed by Sulfatide in BMDM and the Murine Experimental Sepsis Model

**Figure 5:**
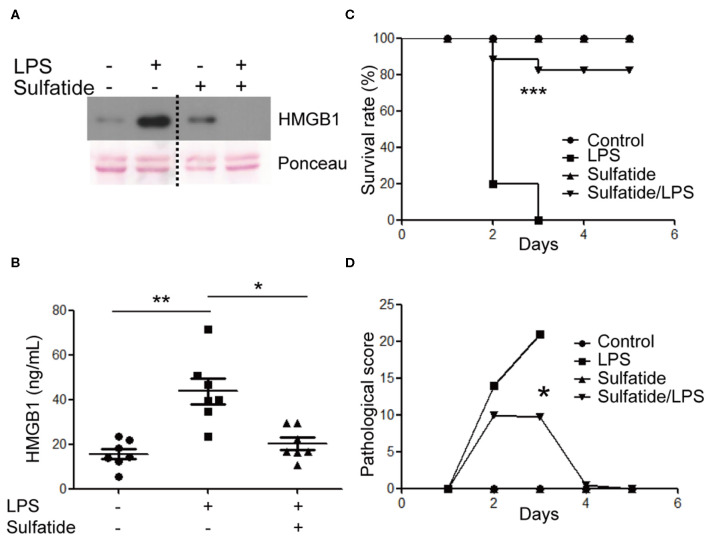
Sulfatide decreases HMGB1 release in mouse BMDMs and the murine experimental sepsis model. **(A)** BMDMs were subjected to vehicle control, LPS 100 ng/mL, and 10 min of 20 μM sulfatide pre-treatment, followed by LPS 100 ng/mL for 24 h. The dotted line indicates where different portion of the identical membrane have been presented together. **(B)** C57BL/6 mice (7 mice per group) were intraperitoneally injected with PBS, LPS, or sulfatide pre-treatment, followed by LPS injection, as discussed in the Methods section. Sera were harvested and prepared for ELISA to measure serum HMGB1 level. **(C)** C57BL/6 mice (5 mice per group) were subjected to a survival test against LPS-induced lethal septic shock. Two independent trials were completed, and the results were pooled for statistical analysis. **(D)** A pathological score was obtained from the mice described in **(C)**. Mice from the second trial were used. *p < 0.01, **p < 0.001, ***p < 0.0001.

The effects of sulfatide in primary cells and *in vivo* murine models were measured. BMDMs of 8 weeks old female C57BL/6 mice were harvested and were subjected to the same stimuli used above (Figure 5A). BMDMs pre-treated with sulfatide showed significantly decreased HMGB1 secretion, compared to cells treated with LPS alone, congruent with data obtained with Raw 263.7 cells. Such conformity led us to induce an experimental septic shock by the means of a sub-lethal dose injection of LPS into the peritoneum of C57BL/6 mice. Measurement of serum HMGB1 level was taken from sera obtained from a total of 18 mice (Figure 5B). Serum HMGB1 level was significantly decreased in the groups pre-treated with sulfatide, compared to groups treated only with LPS (Figure 5B). These results show that sulfatide regulates the release of HMGB1, a late time point cytokine of sepsis, in the murine experimental sepsis model. Such decrease in the serum HMGB1 level is also reflected in the murine model injected with a lethal dosage of LPS, mimicking acute septic shock. Although showing the telltale signs of septic shock (decreased physical activity, shivering etc.), mice pre-injected with sulfatide before LPS injection experienced no death in the population, in contrary to those that received saline pre-injection (Figure 5C). Additionally, to accurately compare the severity of the septic shock and the effect of sulfatide in decreasing its severity, pathological scores were measured every 24 h. Sulfatide pre-injected mice showed similar increase in pathological scores as the mice injected only with LPS for the first 24 h; however, groups that only received LPS injection showed a continuous increase in pathological scores, whereas the scores of the sulfatide pre-injected group plateaued, followed by a decrease in the pathological score (Figure 5D). Generally, sulfatide successfully blocked the LPS-mediated HMGB1 release in sepsis, decreasing the level of serum HMGB1 and preventing severe symptoms and death caused by sepsis.

## Discussion

**Figure 6:**
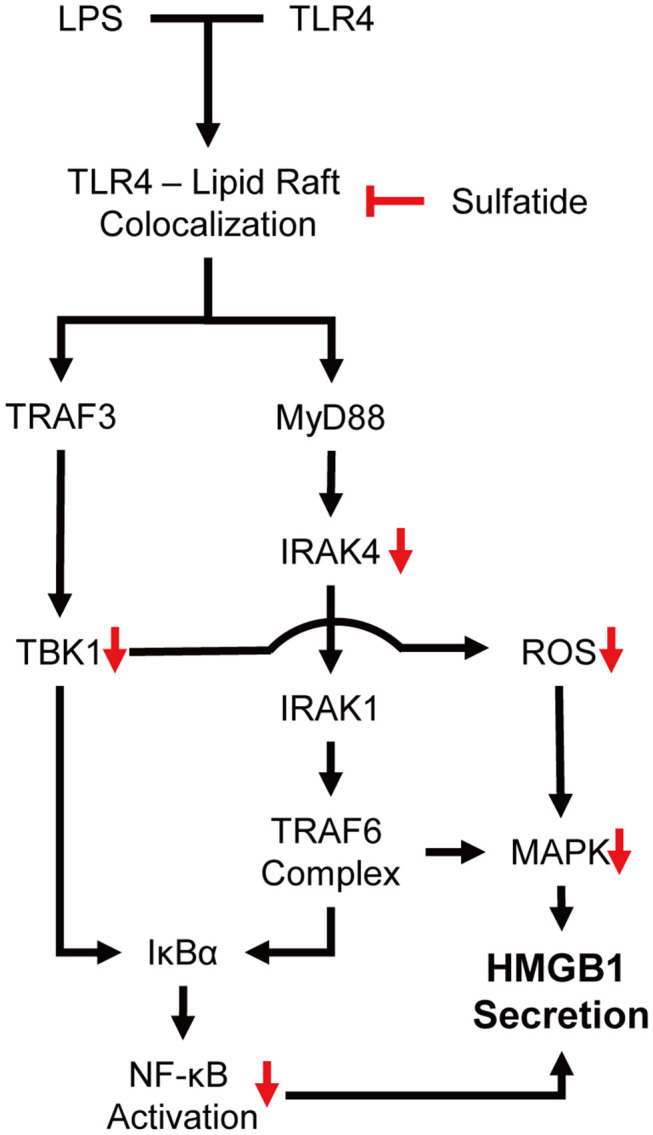
Schematic summary of the proposed mechanism of action.

Our experiments showed sulfatide reducing HMGB1 secretion and cytosolic translocation upon LPS stimulation. Sulfatide decreased the activation of NF-κB translocation into the nucleus, and inhibition of multiple kinases, such as JNK, IRAK4, and TBK1, was also seen throughout the experiment. JNK is a well-known signaling molecule playing a crucial role in cellular stress conditions, and when activated, phosphorylated JNK can also alter the mitochondria to increase its ROS production significantly, creating a positive feedback loop (44). Mice expressing inactive mutant form of IRAK4 were found to be more susceptible to *Listeria monocytogenes* and *Mycobacterium smegmatis* systemic infections due to impaired induction of inducible nitric oxide synthase (iNOS) mRNA (45). Since TBK1 was also involved in mitophagic regulation of mitochondrial physiology and expression of iNOS mRNA during inflammatory assault, paired with the reduction of ROS production, we hypothesized that the inhibitory characteristics of sulfatide may come from the upper hierarchy (46, 47). Further experiments showed sulfatide was hindering the lipid raft–TLR4 interaction, thereby diminishing the TLR4 signaling pathway (Figure 6).

Based on our research, the possibility of exogenous sulfatide as regulator of lipid raft—receptor complex formation may be suggested in clinical scenarios, in addition to the experimental sepsis model provided within. Pathological action of angiotensin II, a potent vasoconstrictor which binds to the AT_1_ receptor, are ascribed to multiple vascular diseases, such as hypertension and secondary cardiac hypertrophy (48). AT_1_ receptors are reported to be associated with lipid rafts (49); thus, sulfatide can be used to alter the lipid composition of the microdomains to deter the pathology in angiotensin II-mediated hypertension patients. Moreover, the immunological synapse, crucial for B/T cell activation, also depends on lipid raft formation (50–53), proposing a potential treatment strategy against autoimmune diseases such as rheumatoid arthritis, Type I diabetes, and multiple sclerosis (54–56) by blocking abnormal B/T cell activation.

Sulfatide, however, has been reported as a possible auto-antigen in multiple sclerosis and experimental autoimmune encephalomyelitis (EAE). Lipid microarrays showed specific antibodies against various lipids in the cerebrospinal fluid, including ones against sulfatide in the murine EAE model and in multiple sclerosis (57, 58). Kanter et al. also reported the increase in disease severity as the mice were immunized with sulfatide and myelin membrane proteins. A further role of sulfatide as a pro-inflammatory molecule in pathogenesis was discovered in autoimmune hepatitis (59). In contrast, the anti-inflammatory roles of sulfatide were also revealed in autoimmune neuritis and asthma, mediated by sulfatide-activated type II NKT cells (37, 60). These reports suggest that sulfatide can be a double-edged sword, depending on the organ and pathological context, and that caution must be taken when attempting to adapt the “natural” form of sulfatide as a potential therapeutic agent. Further studies regarding the mechanism of action of sulfatide in the abovementioned pathologies should be pursued to minimize or ameliorate side effects, possibly by utilizing small molecules mimicking the action of sulfatide.

Our research was able to report the anti-inflammatory effect of sulfatide in the periphery, specify the kinases within the NF-κB and MAPK pathway affected by sulfatide, and elucidate its mechanism of action. Sulfatide, nevertheless, is naturally a mixture of varying lengths of carbon chain backbone; therefore, the sulfatide used in this experiment is close to its natural form but far from being homogenous. Such properties could control the accessibility of sulfatide isoforms to various molecules via steric hindrance and variation in affinity. The composition of sulfatide isoforms has been connected to MS prognosis, enabling physicians to differentiate remitting MS from progressive MS by studying the composition of sulfatide isoforms (61). Although we were able to discover sulfatide hampering the localization of TLR4 and lipid rafts in our research, the specific roles of each component of sulfatide are yet to be discovered. According to the composition sheet provided by the supplier, C24-related isoforms were dominant in the making of sulfatide. This may explain the difference in phenotype between our experiment and others, as we carefully suggest the difference stems from the variability of sulfatide composition, depending on the provider. Isaac et al. has reported the importance of C18 sulfatide in astrocyte functionality (62), whereas many researchers including Buschard et al. and Blomqvist et al. have reported the crucial role of the C16:0 isoform in diabetes mellitus (63, 64). Such reports describing distinct role of various sulfatide components could be used to aid in indirectly understanding the phenotype difference between our group and the others. We sought to specify the isoform solely responsible for the phenotype shown within C24-related isoforms and C18 sulfatide, but to no avail (data not shown). Although we were not able to establish the isoform of sulfatide that is responsible for its properties, we were able to suggest that the mixture of C24-related isoforms and the C18 isoform that mimics the natural composition of sulfatide could also mimic its suppressive phenotype (Supplementary Figure 2). Fine-tuning the composition of specific isoforms by the means of supplementing the patients with sulfatide isoforms as needed may prove to be useful to alter the overall phenotype of sulfatide, further opening its therapeutic potential.

In conclusion, our study showed the effect of sulfatide in suppressing the secretion of HMGB1 under LPS stimulation, and its potential as anti-sepsis treatment. We have also firstly described the mechanism of inhibition where sulfatide inhibits the localization of TLR4 within the lipid microdomains, nullifying LPS-TLR4 signaling cascade. Further investigations regarding the interaction of exogenous sulfatide with lipid microdomains, importance of sulfatide isoform composition in various inflammatory diseases, and in-depth studying of isoform lipid biology are necessary to pursue future therapeutic applications of sulfatide.

## Data Availability Statement

All datasets generated for this study are included in the article/Supplementary Material.

## Ethics Statement

The animal study was reviewed and approved by Institutional Animal Care and Use Committee of the Yonsei Laboratory Animal Research Center.

## Author Contributions

HK performed the immunoblotting and ELISA analysis with regard to HMGB1 secretion and its mechanisms. MH and IP helped in the planning and execution of the animal experiments. CP aided with the overall experiments using BMDMs. MK and IP contributed to the detailed experimental planning. J-SS gave the final approval of the manuscript version and has overseen the project and provided overall guidance for the experimental design. All authors contributed to the article and approved the submitted version.

## Conflict of Interest

The authors declare that the research was conducted in the absence of any commercial or financial relationships that could be construed as a potential conflict of interest.
