# Supplementary material for: Sulfatide Inhibits HMGB1 Secretion by Hindering Toll-Like Receptor 4 Localization Within Lipid Rafts
Source: Front Immunol. 2020 Jun 23;11:1305. doi: 10.3389/fimmu.2020.01305 (PMC7324676; doi:10.3389/fimmu.2020.01305)
Supplement: Supplementary file 1 [file Table_1.docx]

Supplementary Materials

#
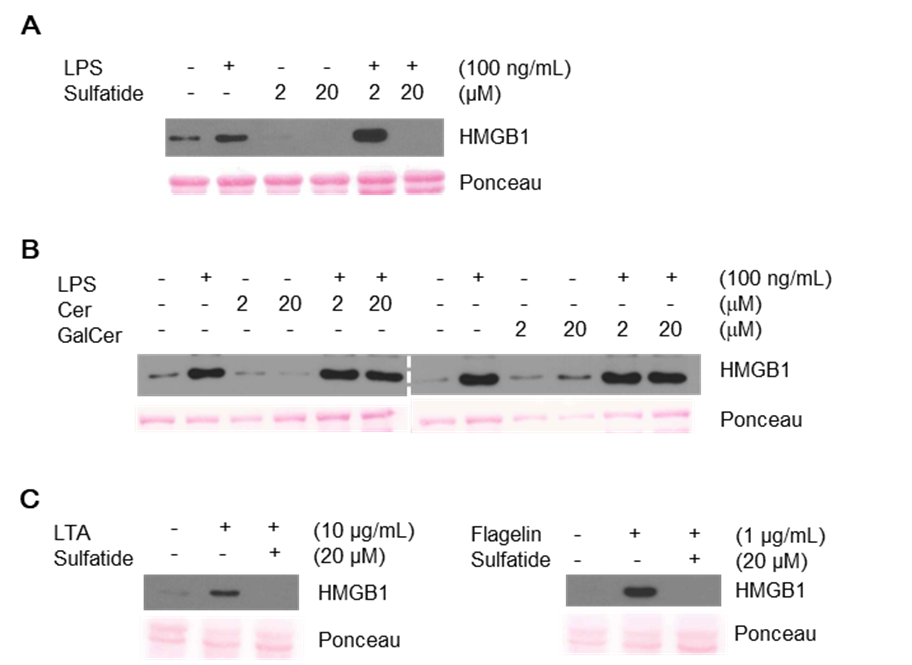
Supplementary Figures

**Supplementary Figure 1.** **(A)** Culture media were analyzed by immunoblot for HMGB1 secretion. Cells were treated with vehicle control (PBS with DMSO), LPS 100 ng/mL, or 10 minutes of sulfatide 20 μM pre-treatment, followed by LPS 100 ng/mL. **(B)** Treatment with galactosylceramide or ceramide was done before LPS or vehicle controls were applied to Raw 264.7 cells. Cells received the abovementioned stimuli and were cultured for 24 hours. Culture media were concentrated and analyzed by immunoblotting as described in the Methods section. **(C)** The effect of sulfatide on other extracellular TLRs (TLR2 and 5) was accessed.


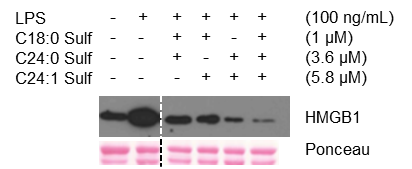


**Supplementary Figure 2.** Raw 264.7 cells received combinations of sulfatide components before LPS stimulation or vehicle control treatment. Different concentrations of sulfatide components used are representative of composition ratio of the sulfatide mixture, provided by the supplier. Culture media were then harvested 24 hours after treatment, concentrated, and analyzed by immunoblotting as described in the Methods section. The dotted line indicates where different portion of the identical membrane have been presented together.
